# Dietary *Hermetia illucens* Larvae Meal Improves Growth Performance and Intestinal Barrier Function of Weaned Pigs Under the Environment of Enterotoxigenic *Escherichia coli* K88

**DOI:** 10.3389/fnut.2021.812011

**Published:** 2022-01-18

**Authors:** Qingsong Tang, E. Xu, Zhikang Wang, Mingfei Xiao, Shuting Cao, Shenglan Hu, Qiwen Wu, Yunxia Xiong, Zongyong Jiang, Fengying Wang, Geling Yang, Li Wang, Hongbo Yi

**Affiliations:** ^1^State Key Laboratory of Livestock and Poultry Breeding, Ministry of Agriculture Key Laboratory of Animal Nutrition and Feed Science in South China, Guangdong Key Laboratory of Animal Breeding and Nutrition, Maoming Branch, Guangdong Laboratory for Lingnan Modern Agriculture, Institute of Animal Science, Guangdong Academy of Agricultural Sciences, Guangzhou, China; ^2^Key Laboratory of Animal Genetics, Breeding and Reproduction in the Plateau Mountainous Region, Institute of Animal Nutrition and Feed Science, College of Animal Science, Ministry of Education, Guizhou University, Guiyang, China; ^3^Guangzhou AnRuiJie Environmental Protection Technology Co., Ltd., Guangzhou, China

**Keywords:** *H. illucens*, growth performance, intestinal barrier function, microbiota, weaned pigs, histone acetylation

## Abstract

The aim of this study was to evaluate the effect of *Hermetia illucens* larvae meal (HI) on the growth performance and intestinal barrier function of weaned pigs. To achieve this, 72 weaned pigs [28-day-old, 8.44 ± 0.04 kg body weight (BW)] were randomly assigned to three dietary treatments: basal diet (negative control, NC), zinc oxide-supplemented diet (positive control, PC), and HI-supplemented diet [100% replacement of fishmeal (FM), HI], for 28 days in the presence of enterotoxigenic *Escherichia coli* (ETEC). The results showed that HI and PC increased (*p* < 0.05) the average daily gain (ADG) and average daily feed intake (ADFI) of weaned pigs from day 1 to 14, and decreased diarrhea incidence from day 1 to 28. Additionally, HI increased (*p* < 0.05) claudin-1, occludin, mucin-1 (*MUC-1*), and MUC-2 expression, goblet cell number, and secretory immunoglobulin A (sIgA) concentration in the intestine of weaned pigs. Compared with NC, HI downregulated (*p* < 0.05) interleukin-1β (*IL-1*β) and *IL-8* expression, and upregulated *IL-10*, transforming growth factor-β (*TGF-*β), antimicrobial peptide [porcine β defensin 1 (*pBD1*), *pBD2*, protegrin 1-5 (*PG1-5*)] expression in the jejunum or ileum. Moreover, HI decreased (*p* < 0.05) toll-like receptor 2 (TLR2), phosphorylated nuclear factor-κB (p-NF-κB), and phosphorylated mitogen-activated protein kinase (p-MAPK) expression, and increased sirtuin 1 (SIRT1) expression in the ileum. Additionally, HI increased histone deacetylase 3 (HDAC3) expression and acetylation of histone 3 lysine 27 (acH3k27) in the ileum. Furthermore, HI positively influenced the intestinal microbiota composition and diversity of weaned pigs and increased (*p* < 0.05) butyrate and valerate concentrations. Overall, dietary HI improved growth performance and intestinal barrier function, as well as regulated histone acetylation and TLR2-NF-κB/MAPK signaling pathways in weaned pigs.

## Introduction

Fishmeal (FM) is an important protein source in piglet nutrition; however, marine overfishing has led to soaring FM prices and reduced its availability (1). In 2019, ~1.420 million tons of FM were imported by China, with a local supply of only 0.699 million tons, indicating that China is extremely dependent on importation to meet its FM demand. Therefore, to reverse this trend, there is a need for effective, safe, and sustainable alternatives to FM, one of which is *Hermetia illucens* larvae meal (HI).

*Hermetia illucens* larvae is a common insect with high protein content (37–44.6%) and an essential amino acid profile similar to that of fishmeal (1–3). Importantly, *H. illucens* larvae are rich in antimicrobial peptides (AMPs) and medium-chain fatty acids, which play important roles in regulating animal intestinal microbiota and intestinal immunity (4, 5). Studies have reported improved growth performance in Chinese soft-shelled turtles, chickens, and fattening pigs fed diets containing appropriate levels of *H. illucens* larvae as the protein source (6–8). Additionally, HI has been shown to promote the expression of mucins (MUCs) and tight junction (TJ) proteins, and improve intestinal barrier function in juvenile barramundi, broilers, and fattening pigs (9–11).

Furthermore, some studies have shown that HI improves the intestinal microflora and metabolism, and regulates immune homeostasis in fish, broilers, and fattening pigs (11–13). A recent study reported that feeding 2% HI (replacing 50% of dietary FM) improved intestinal microflora and intestinal immune homeostasis in weaned pigs (14), which is probably through the activities of AMPs. Research findings suggest that the expression of AMPs depends on histone acetylation. Histone deacetylase (HDAC) and sirtuin 1 (SIRT1) regulates the modification mode of histone H3 [acetylation of histone lysine 9 (acH3k9), acetylation of histone 3 lysine 27 (acH3k27), and phosphorylation of histone H3 at serine s10 (pH3s10)] in the promoter region of antimicrobial peptides, which specifically increases the expression of intestinal AMPs and enhances their ability against bacterial infections (15–17). However, it is unclear whether HI regulates the expression of AMPs through histone acetylation, improving the intestinal microbiota and intestinal immune homeostasis of weaned pigs. Moreover, although studies have examined the effect of HI diet on aquatic animals, poultry, and fattening pigs, studies on weaned pigs are limited.

Enterotoxigenic *Escherichia coli* (ETEC) K88 is one of the main causes of diarrhea in weaned pigs, and is strongly associated with retarded growth performance, intestinal barrier dysfunction, and microbiota disorders (18, 19). However, the effect of HI on weaned pigs in the environment of ETEC K88 has not been reported to date. Therefore, the aim of this study was to examine the effect of HI on the growth performance, intestinal barrier function, and intestinal microbiota of weaned pigs in the environment of ETEC K88.

## Materials and Methods

### Preparation of HI

HI was provided by Guangzhou AnRuiJie Environmental Protection Technology Co., Ltd. (Guangzhou, China). The prepupae were dried at 80°C for 30 min and air-dried, crushed into powder, and passed through a 1.0 mm sieve as described previously (11). The DM (GB/T 6435-2014), ash (GB/T 6438-2007), CP (GB/T 6432-1994), EE (GB 5009.6-2016), total P (GB/T 6437-2018), Ca (GB 5009.268-2016), and amino acid (GB/T 18246-2000) content of HI was determined by Guangzhou HuiBiao Testing Technology Center, as shown in Table 1.

**Table 1:** Nutritional composition of the *Hermetia illucens* larvae meal.

**Nutritional composition**	**Content**
DM, %	93.3
Ash, %	17.6
CP, %	37.91
EE, %	31.2
Total P, %	0.83
Ca, %	4.39
**Essential amino acids**	
Lys	1.88
Met	0.40
Met + Cys	0.50
Ile	1.16
Leu	2.02
Trp	0.36
Val	1.67
Thr	1.27
Arg	1.65
Phe	1.05
His	0.89
**Non-essential amino acids**	
Ala	2.05
Asp	2.86
Glu	4.24
Gly	1.61
Ser	1.21
Tyr	1.83
Pro	2.07

### Experimental Procedure and Sample Collection

A total of 72 crossbred pigs [Duroc × (Landrace × Yorkshire)] with initial body weight (BW) of 8.44 ± 0.04 kg were weaned at an average age of 28 days and randomly allocated to three treatments (six replicate pens per treatment and four weaned pigs per pen) in a complete randomized experimental design. Pigs were fed a basal diet (negative control, NC), and basal diet supplemented with 1,445 mg zinc/kg of zinc oxide (positive control, PC) or basal diet with 3% FM replaced with HI. The nutritional composition of the basal diets met the requirements recommended by the National Research Council (20) and are shown in Table 2. An ultra-low volume sprayer (Yitaizheng 211) was used to evenly spray 6 × 10^8^ CFU/mL ETEC K88 bacterial solution in a 60 m^2^ environment. The spray volume of the entire environment was 2 L at a constant flow rate. The pigsty was not cleaned and disinfected during the entire trial period. Environmental samples were collected on day 10 of the experiment from the leakage dung plate, water dispenser, and aisle in the pig house and stored in 10 mL preservation tubes. The number of *E. coli* was measured using eosin-methylene blue agar medium. The results showed that the number of *E. coli* on the leakage dung plate, water dispenser, and aisle, was 5.19 × 10^8^ CFU/m^2^, 4.79 × 10^4^ CFU/piece, and 1.78 × 10^8^ CFU/m^2^, respectively. The experimental pigs had *ad libitum* access to water and assigned diet. When the feeding trial ended, 18 pigs (six per group) were euthanized by intravenous injection of sodium pentobarbital solution (40 mg/kg body weight) and segments of the middle jejunum and distal ileum were collected and fixed in 4% paraformaldehyde for intestinal morphology analysis. Segments of the middle jejunum and distal ileum, and colonic contents were snap-frozen in liquid N_2_ and stored at −80°C for further analysis.

**Table 2:** Composition and nutrient levels of diets (as-fed basis, %).

**Ingredients**	**NC**	**PC**	**HI**
Corn	28.45	28.45	26.55
Extruded corn	10.00	10.00	10.00
Soybean flour processing	14.00	14.00	14.00
Fermented soybean meal	11.50	11.50	11.50
Soybean meal	7.00	7.00	9.00
Fishmeal	3.00	3.00	–
*Hermetia illucens* larvae meal	–	–	3.00
Low protein whey powder	15.00	15.00	15.00
Whey protein concentrate	1.00	1.00	1.00
Soybean oil	1.00	1.00	1.00
Sucrose	3.00	3.00	3.00
CaHPO_4_	0.20	0.20	0.20
NaCl	0.25	0.25	0.25
CaHPO_4_	0.60	0.60	0.85
Calcium citrate	1.85	1.85	1.50
*L*-Lys·HCl	0.55	0.55	0.55
*DL*-Met	0.15	0.15	0.15
*L*-Thr	0.20	0.20	0.20
*L*-Try	0.05	0.05	0.05
*L*-Val	0.10	0.10	0.10
Choline chloride	0.20	0.20	0.20
TiO_2_	0.40	0.40	0.40
Premix^a^	1.50	1.50	1.50
Total	100.00	100.00	100.00
**Nutrient levels^b^**			
ME, MJ/kg	14.34	14.34	14.35
CP	21.03	21.07	20.98
EE	5.89	5.68	6.64
SID Lys	1.44	1.44	1.43
SID Met	0.44	0.44	0.42
SID Thr	0.85	0.85	0.85
SID Trp	0.26	0.26	0.27
SID Val	0.92	0.92	0.93
SID Ile	0.76	0.76	0.76
Ca	0.83	0.83	0.80
STTD P	0.35	0.35	0.35
Total P	0.60	0.57	0.54

a *The premix provided per kg of the diet: VA 12,400 IU, VD3 2,800 IU, VE 30 IU, VK 5 mg, VB_1_ 3 mg, VB_2_ 10 mg, niacin 40 mg, pantothenic acid 15 mg, folic acid 1 mg, VB_6_ 8 mg, biotin 0.08 mg, VB_12_ 40 μg, Fe(FeSO_4_·H_2_O)120 mg, Cu(CuSO_4_·5H_2_O) 16 mg, Mn(MnSO_4_·H_2_O) 70 mg, Zn(ZnSO_4_·H_2_O) 100 mg, I(CaI_2_O_6_) 0.7 mg, Se(Na_2_SeO_3_) 0.48 mg*.

b *CP, EE, Ca, and TP were measured values, whereas the others were calculated values*.

### Bacterial Culture

The ETEC K88 used in this experiment was a strain preserved in our laboratory. ETEC K88 stored in glycerol medium at −80°C was resuscitated, poured into a 25 mL centrifuge tube, and sterilized Luria-Bertani medium was added and the tubes were cultured in an incubator at 37°C for 12 h. Thereafter, the entire liquid was poured into a conical bottle containing 1 L sterilized Luria-Bertani medium, placed in a shaker (250 rpm), and cultured at 37°C for 24 h. Thereafter, the concentration of ETEC K88 was determined using enzyme-labeling instrument, and the solution was sub-packaged at −20°C. Before use, 200 mL of the solution was diluted 10-fold with 0.9% normal saline.

### Growth Performance and Diarrhea Incidence

To evaluate the growth performance of the pigs, the weaned pigs were fasted for 12 h and weighed at 08:00 on days 1, 15, and 29 of the experiment. The average daily gain (ADG), average daily feed intake (ADFI), and feed/gain ratio (F/G) were calculated at the end of the experiment. To determine the incidence and severity of diarrhea, the feces of the pigs were observed twice daily throughout the study and scored: 0, normal; 1, soft stool; 2, mild diarrhea; and 3, severe diarrhea, with a score ≥ 2 considered as diarrhea (21). The formula for calculating the diarrhea incidence and diarrhea index was: diarrhea incidence (%) = Σ (the number of pigs with diarrhea per pen × days of diarrhea)/(total number of pigs × number of experimental days) × 100; diarrhea index = Σ (diarrhea score × number of pigs scored for diarrhea × days of diarrhea score)/(total number of pigs × number of experimental days).

### Histomorphological and Histological Analysis

Fixed jejunal and ileal segments were dehydrated, embedded in paraffin, cut into 5 μm thick slices, and stained with hematoxylin and eosin (HE) staining. Another jejunal slice was stained with periodic acid-Schiff (PAS) as described previously (22). Subsequently, slides were scanned using Pannoramic DESK (3D Histech, Hungary), and all measurements were conducted using the associated Pannoramic Scanner software. A minimum of 10 straight and integrated villi and their associated crypts were measured in HE section images. The number of goblet cells and mucus layer thickness was measured in at least three microscopic fields (original magnification × 100) in the PAS section images.

### RNA Isolation and Quantitative Real-Time PCR

Total RNA from the jejunum and ileum was extracted using TRIzol reagent (Invitrogen, Carlsbad, CA, USA). RNA purity and concentration were assessed using a NanoDrop 1000 spectrophotometer (Thermo Fisher Scientific, Waltham, USA). Thereafter, 1 μg of RNA was reverse transcribed into cDNA using a PrimeScriptTM II 1st Strand cDNA Synthesis Kit (Takara, Tokyo, Japan), according to the manufacturer's instructions. Quantitative real-time PCR was performed using a CFX Connect Detection System (Bio-Rad, Hercules, CA, USA). The reaction mixture (10 μL) comprised 5 μL iTaq Universal SYBR Green Supermix, 0.5 μL forward primer (10 μm), 0.5 μL reverse primer (10 μm) and 4 μL 10-fold diluted cDNA template. The target genes examined in this study and sequences for the target gene and housekeeping gene (β-actin) are listed in Table 3. The mRNA expression data of the target genes were calculated using the 2^−ΔΔCt^ method, and target gene expression in the NC group was set at 1.0.

**Table 3:** Primers for the real-time PCR analysis.

**Gene**	**Sequence (5^**′**^-3^**′**^)**	**Size (bp)**	**Accession number**
*IL-1β*	F: CTCCAGCCAGTCTTCATTGTTC R: TGCCTGATGCTCTTGTTCCA	230	NM214055.1
*IL-6*	F: TGGCTACTGCCTTCCCTACC R: CAGAGATTTTGCCGAGGATG	132	NM_001252429.1
*IL-8*	F: TTCGATGCCAGTGCATAAATA R: CTGTACAACCTTCTGCACCCA	176	NM_213867.1
*IL-10*	F: GCTGAAGACCCTCAGGCTGA R: TTGCTCTTGTTTTCACAGGGC	66	HQ026020.1
*TGF-β*	F: ACGTGGAGCTATACCAGAAATACAG R: ACAACTCCGTGACATCAAAGG	111	NM_214015.1
*TNF-α*	F: CACGCTCTTCTGCCTACTGC R: GTCCCTCGGCTTTGACATT	164	NM_214022.1
*ZO-1*	F: AGCCCGAGGCGTGTTT R: GGTGGGAGGATGCTGTTG	147	XM_013993251
*Occludin*	F: GCACCCAGCAACGACAT R: CATAGACAGAATCCGAATCAC	144	XM_005672525
*Claudin-1*	F: ACGGCCCAGGCCATCTAC R: TGCCGGGTCCGGTAGATG	221	AJ318102.1
*MUC-1*	F: ACACCCATGGGCGCTATGT R: GCCTGCAGAAACCTGCTCAT	68	XM_021089730.1
*MUC-2*	F: CTGCTCCGGGTCCTGTGGGA R: CCCGCTGGCTGGTGCGATAC	100	XM_007465997.1
*pBD1*	F: TCCTTGTATTCCTCCTCA R: ACACGCCTTTATTCCTTA	93	NM_213838.1
*pBD2*	F: CCAGAGGTCCGACCACTACA R: GGTCCCTTCAATCCTGTTGAA	88	AY506573.1
*PG1-5*	F: GTAGGTTCTGCGTCTGTGTCG R: CAAATCCTTCACCGTCTACCA	273	XM_005669497.2
*PR-39*	F: CAAGGCCACCTCCGTTTT R: CCACTCCATCACCGTTTTCC	103	NM_214450.1
*NOD1*	F: ACCGATCCAGTGAGCAGATA R: AAGTCCACCAGCTCCATGAT	140	NM_001114277.1
*NOD2*	F: CCTTTTGAAGATGCTGCCTG R: GATTCTCTGCCCCATCGTAG	100	NM_001105295.1
*TLR2*	F: TCACTTGTCTAACTTATCATCCTCTTG R: TCAGCGAAGGTGTCATTATTGC	162	NM_213761.1
*TLR4*	F: GCCATCGCTGCTAACATCATC R: CTCATACTCAAAGATACACCATCGG	108	NM_001113039.1
*β-actin*	F: CACGCCATCCTGCGTCTGGA R: AGCACCGTGTTGGCGTAGAG	380	XM_003124280.4

*TGF-β, transforming growth factor-β; ZO-1, zonula occludens-1; pBD, porcine beta defensin; PG1-5, protegrin 1-5; PR-39, proline-arginine rich 39; NOD, nucleotide-binding oligomerization domain; TLR, toll-like receptor*.

### ELISA

Approximately 100 mg of intestinal tissue sample was added to 900 μL of 0.86% normal saline, homogenized with a tissue homogenizer, and centrifuged at 3,500 × *g* at 4°C for 10 min; the supernatant was used to determine the level of secretory immunoglobulin A (sIgA) in the jejunum and ileum using a porcine ELISA kit (Jiancheng, Nanjing, China).

### Western Blot

Approximately 100 mg of intestinal tissue sample was placed in 1 mL of RIPA buffer containing 1% protease inhibitor cocktail and 1% phosphatase inhibitor, left on ice for 30 min, and centrifuge at 10,000 × *g* for 5 min. The concentration of protein in the supernatant was determined using a BCA protein assay kit (Thermo Fisher Scientific, Wilmington, USA). Equivalent amounts of protein were diluted with 5 × loading buffer (Biosharp, Hefei, China) to the same amount of protein and denatured at 100°C for 8 min. Approximately 30 μg of protein was separated using 7.5 or 10% SDS-PAGE, transferred onto polyvinylidene fluoride membrane, blocked using 5% bovine serum albumin, incubated overnight at 4°C with the primary antibodies, followed by incubation for 1 h at room temperature with secondary antibodies. For phosphorylated proteins, the primary and secondary antibodies [(phosphorylated nuclear factor-κB (p-NF-κB), phosphorylated mitogen-activated protein kinase (p-MAPK)] from a western blot membrane were striped and then incubated with the antibodies (NF-κB, MAPK) for total protein detection. Primary antibodies against NF-κB p65, phosphorylated NF-κB p65, MUC-2, occludin, claudin 1, pH3s10, acH3k9, acH3k27, histone H3, HDAC3, HDAC7, and SIRT1 were purchased from Abcam; p38 mitogen-activated protein kinase (MAPK) and phosphorylated p38 MAPK were purchased from Cell Signaling Technology; zonula occludens-1 (ZO-1) was purchased from Thermo Fisher Scientific, and β-actin was purchased from Abcam. The blots were detected using Clarity Western ECL Substrate (Bio-Rad, Hercules, CA, USA). The protein band intensities were measured using ImageJ software (National Institutes of Health, MD, USA).

### Microbial Composition Analysis

Total DNA was extracted from each sample of colonic content using the CTAB method, and the purity and yield were quantified using a NanoDrop spectrophotometer (Thermo Fisher Scientific, Wilmington, USA) and agarose gels. The V3-V4 regions of the 16S rRNA genes were amplified using the forward primer 341F (5′-CCTAYGGGRBGCASCAG-3′) and the reverse primer 806R (5′-GGACTACNNGGGTATCTAAT-3′). The PCR products were recovered and purified using the GeneJETTM Gel Extraction Kit (Thermo Fisher Scientific, Wilmington, USA). Validated libraries were sequenced on the NovaSeq6000 platform provided by Novogene (Beijing, China).

### Determination of SCFA Concentrations

Gas chromatography-mass spectrometry (GC-MS) TRACE 1310-ISQ was used to measure the concentration of short-chain fatty acids (SCFAs) in colonic contents. Briefly, the colon contents were weighed and added to 50 μL of 15% phosphoric acid, 100 μL of 125 μg/mL internal standard, and 400 μL of ether. The supernatant was collected after centrifugation at 12,000 × *g* for 10 min at 4°C, and the concentration of SCFAs was measured using GC-MS analysis, as described previously (23).

### Statistical Analysis

All experimental data were analyzed using SPSS software package (version 26.0; SPSS Inc., Chicago, IL, USA). Data obtained during the study were normalized using Shapiro-Wilk normality test. Data were analyzed using one-way ANOVA and differences among treatment groups were determined using Duncan's multiple range *post-hoc* test. All data were expressed as mean ± SEM, and statistical significance was set at *p* < 0.05.

## Results

### Effect of *H. illucens* on the Growth Performance and Diarrhea Incidence in Weaned Pigs

**Figure 1:**
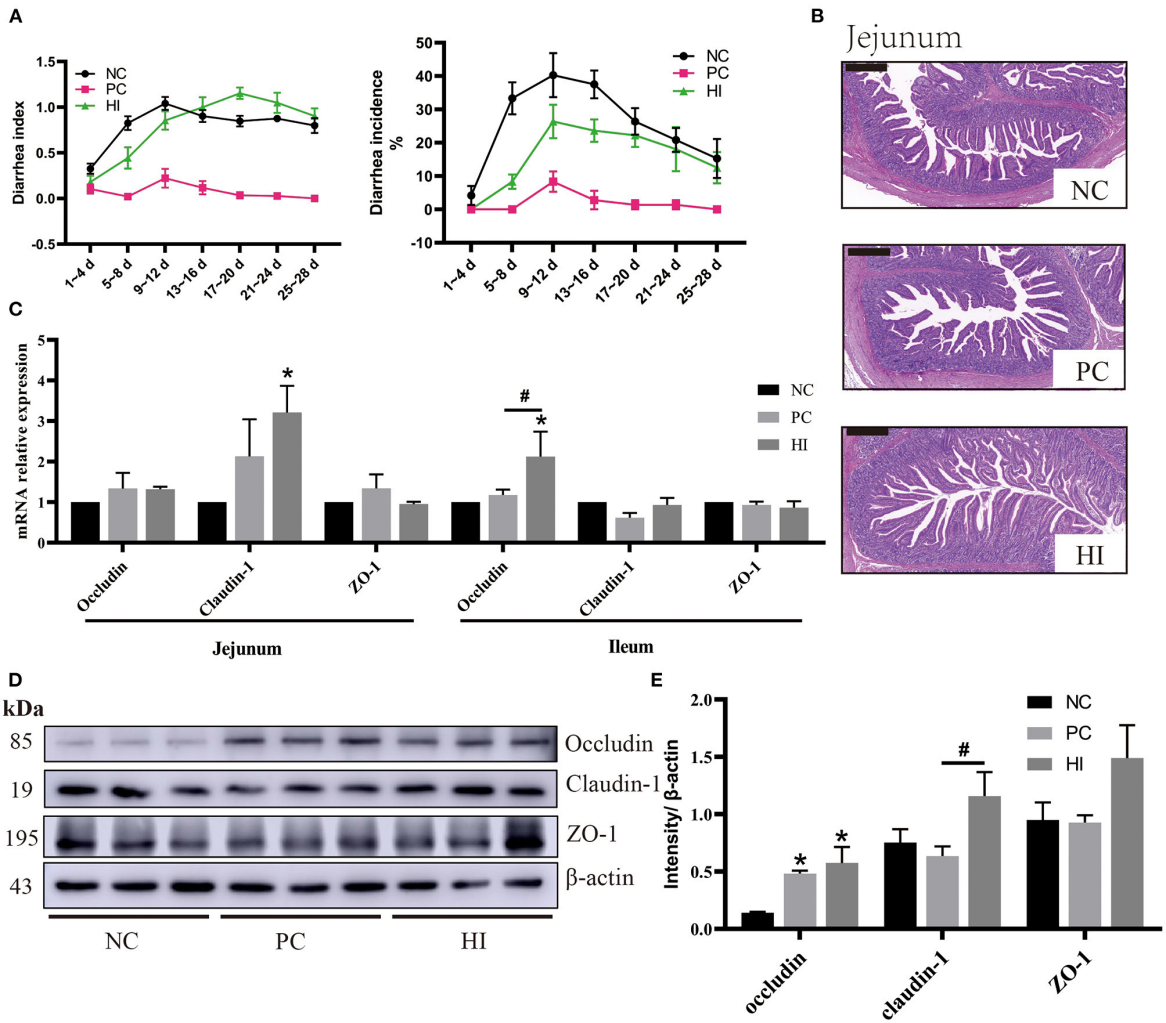
Effect of dietary *Hermetia illucens* larvae on diarrhea incidence, intestinal morphology, and TJ protein expression in weaned pigs. **(A)** Diarrhea incidence and diarrhea index of weaned pigs. **(B)** Representative images of jejunal HE stains (scale bar, 500 μm). **(C)** Relative mRNA expression levels of *occludin, claudin-1*, and *ZO-1* in the jejunum and ileum were determined using real-time PCR. **(D)** Relative protein expression levels of occludin, claudin-1, and ZO-1 in the ileum were measured using western blot. Densitometric values were normalized to those of β-actin. **(E)** Statistical analysis of the data in D. All data are expressed as the mean ± SEM (protein expression data *n* = 3, other data *n* = 6). *p < 0.05 compared with NC, ^#^*p* < 0.05 HI compared with PC.

Compared with the NC group, there was an increase (*p* < 0.05) in the BW of pigs in HI and PC groups on day 14. From day 1 to 14 of the experiment, there was an increase in the ADG and ADFI of pigs in the HI and PC groups compared with those of pigs in the NC group (Table 4). From day 15 to 28 and day 1 to 28 of the experiment, there was an increase (*p* < 0.05) in the ADFI of pigs in the PC group compared with those of pigs in the NC group. The diarrhea incidence of the piglets during the experimental period is shown in Figure 1A. Compared with the NC group, there was a decrease (*p* < 0.05) in diarrhea incidence in pigs in the PC and HI groups during the three periods examined (days 1–14, 15–28, and 1–28). However, pigs in the PC group had lower diarrhea incidence (*p* < 0.05) compared with pigs in the HI group during the three periods (Table 4). Furthermore, there was a decrease (*p* < 0.05) in the diarrhea index of pigs in the PC and HI groups compared with those of pigs in the NC group during 1–14 days and 1–28 days of the study. However, pigs in the PC group had lower diarrhea index than those in the HI and NC groups during 15–28 days of the study.

**Table 4:** Effects of dietary *Hermetia illucens* larvae meal on growth performance and diarrhea incidence in weaned pigs.

**Items**	**NC**	**PC**	**HI**	**SEM**	***p*-value**
Day 1 BW, kg	8.42	8.42	8.47	0.04	0.892
Day 14 BW, kg	11.76^b^	12.81^a^	12.61^a^	0.18	0.026
Day 28 BW, kg	20.61	22.68	21.98	0.45	0.161
**Days 1–14**					
ADG, g	238.81^b^	313.81^a^	295.47^a^	12.10	0.019
ADFI, g	341.99^b^	428.89^a^	402.42^a^	13.89	0.021
G/F	0.70	0.74	0.73	0.02	0.634
Diarrhea incidence, %	28.17^a^	1.98^c^	15.48^b^	2.95	<0.01
Diarrhea index	0.76^a^	0.13^c^	0.57^b^	0.07	<0.01
**Days 15–28**					
ADG, g	631.55	704.52	669.76	22.24	0.433
ADFI, g	852.32^b^	1,046.32^a^	905.32^ab^	38.03	0.092
G/F	0.75	0.68	0.75	0.02	0.328
Diarrhea incidence, %	32.76^a^	2.30^c^	20.11^b^	3.55	<0.01
Diarrhea index	1.24^a^	0.04^b^	1.17^a^	0.14	<0.01
**Days 1–28**					
ADG, g	435.18	509.17	482.62	15.76	0.153
ADFI, g	597.16^b^	737.60^a^	653.87^ab^	22.94	0.031
G/F	0.74	0.70	0.74	0.02	0.600
Diarrhea incidence, %	30.47^a^	2.14^c^	17.80^b^	3.09	<0.01
Diarrhea index	1.00^a^	0.08^c^	0.87^b^	0.10	<0.01

*n = 6 replicates per treatment*.

*NC, basal diet; PC, basal diet supplemented with 1,445 mg zinc/kg of zinc oxide; HI, 100% replacement of FM in basal diet with H. illucens larvae meal*.

a, b *Mean values with different superscript letters across rows are significantly different (p < 0.05)*.

### Effect of HI on the Intestinal Morphology of Weaned Pigs

Compared with the NC and PC groups, there was an increase (*p* < 0.05) in jejunal villi height and ratio of villus height to crypt depth (V/C) of pigs in the HI treatment group. Additionally, compared with the NC group, there was a decrease (*p* < 0.05) in the jejunal crypt depth of pigs in HI group (Table 5; Figure 1B).

**Table 5:** Effect of dietary *Hermetia illucens* larvae on intestinal morphology in weaned pigs.

**Items**	**NC**	**PC**	**HI**	**SEM**	***p*-value**
**Jejunum**					
Villi height/μm	488.43^b^	470.82^b^	567.03^a^	16.38	0.027
Crypt depth/μm	267.09^a^	247.50^ab^	217.53^b^	8.49	0.045
Ratio of villi height to crypt depth	1.88^b^	1.90^b^	2.62^a^	0.11	0.003
**Ileum**					
Villi height/μm	385.69	371.31	381.62	15.52	0.935
Crypt depth/μm	187.79	179.72	189.16	7.62	0.875
Ratio of villi height to crypt depth	2.10	2.12	2.05	0.10	0.969

*n = 6 replicates per treatment*.

*NC, basal diet; PC, basal diet supplemented with 1,445 mg zinc/kg of zinc oxide; HI, 100% replacement of FM in basal diet with H. illucens larvae meal*.

a, b *Mean values with different superscript letters across rows are significantly different (p < 0.05)*.

### Effect of *H. illucens* on the Expression of TJ Protein in the Intestines of Weaned Pigs

Compared with the NC group, there was an increase (*p* < 0.05) in *claudin-1* expression in the jejunum of pigs in the HI group (Figure 1C). In addition, there was an increase (*p* < 0.05) in *occludin* expression in the ileum of pigs in the HI group compared with those of pigs in the NC and PC groups. Furthermore, compared with the NC group, there was an increase (*p* < 0.05) in occludin protein expression in the ileum of pigs in the HI and PC groups. Moreover, there was an increase (*p* < 0.05) in claudin-1 protein expression in the ileum of pigs in HI group compared with those of pigs in the PC and NC groups (Figures 1D,E).

### Effect of *H. illucens* on the Expression of Mucins in the Intestines of Weaned Pigs

**Figure 2:**
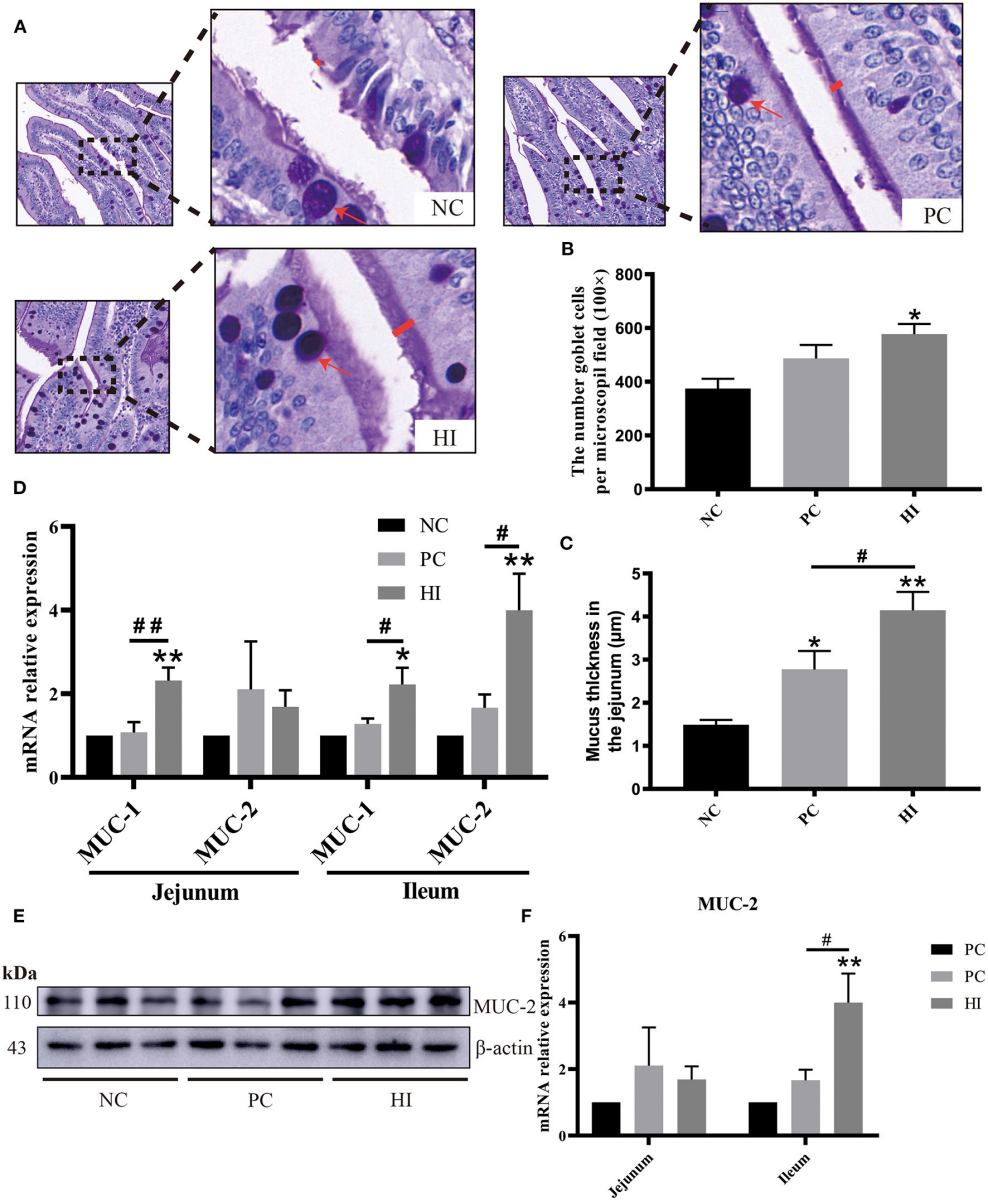
Effect of dietary *Hermetia illucens* larvae on mucin secretion in weaned pigs. **(A)** Representative images of jejunal PAS stains. The number of goblet cells **(B)** per microscopic field (original magnification × 100) and mucus layer thickness **(C)** in the jejunum of weaned pigs were determined. **(D)** Real-time quantitative PCR was performed to determine the relative mRNA expression levels of *MUC-1* and *MUC-2* in the jejunum and ileum. **(E)** Relative protein expression of MUC-2 in the ileum determined using western blot. Densitometric values were normalized to those of β-actin. **(F)** Statistical analysis of the data in E. All data are expressed as the mean ± SEM (protein expression data *n* = 3, other data *n* = 6). *p < 0.05, **p < 0.01 compared with NC, ^#^*p* < 0.05, ^##^*p* < 0.01 HI compared with PC.

Compared with the NC group, there was an increase (*p* < 0.05) in the number of goblet cells in the jejunum of pigs in the HI group (Figures 2A,B). In addition, there was an increase (*p* < 0.01) in mucus layer thickness of the jejunum of pigs in the HI group compared with that of pigs in the NC and PC groups. However, the mucus layer thickness of the jejunum of pigs in the PC group was higher (*p* < 0.05) than those of pigs in the NC group (Figure 2C). Based on the changes in the number of goblet cells and mucus layer thickness of the jejunum, the expression profiles of *MUC-1* and *MUC-2* in the jejunum and ileum were examined. Compared with the NC and PC groups, there was an increase in *MUC-1* expression in the jejunum (*p* < 0.01) and ileum (*p* < 0.05) of pigs in the HI group (Figure 2D). Similarly, there was an increase (*p* < 0.05) in *MUC-2* expression in the ileum of pigs in the HI group compared with those of pigs in the PC and NC groups. Furthermore, western blot analysis showed there was an increase (*p* < 0.05) in the expression of MUC-2 protein in the ileum of pigs in the HI group compared with those of pigs in the NC group (Figures 2E,F).

### Effect of *H. illucens* on the Intestinal Immunity in Weaned Pigs

**Figure 3:**
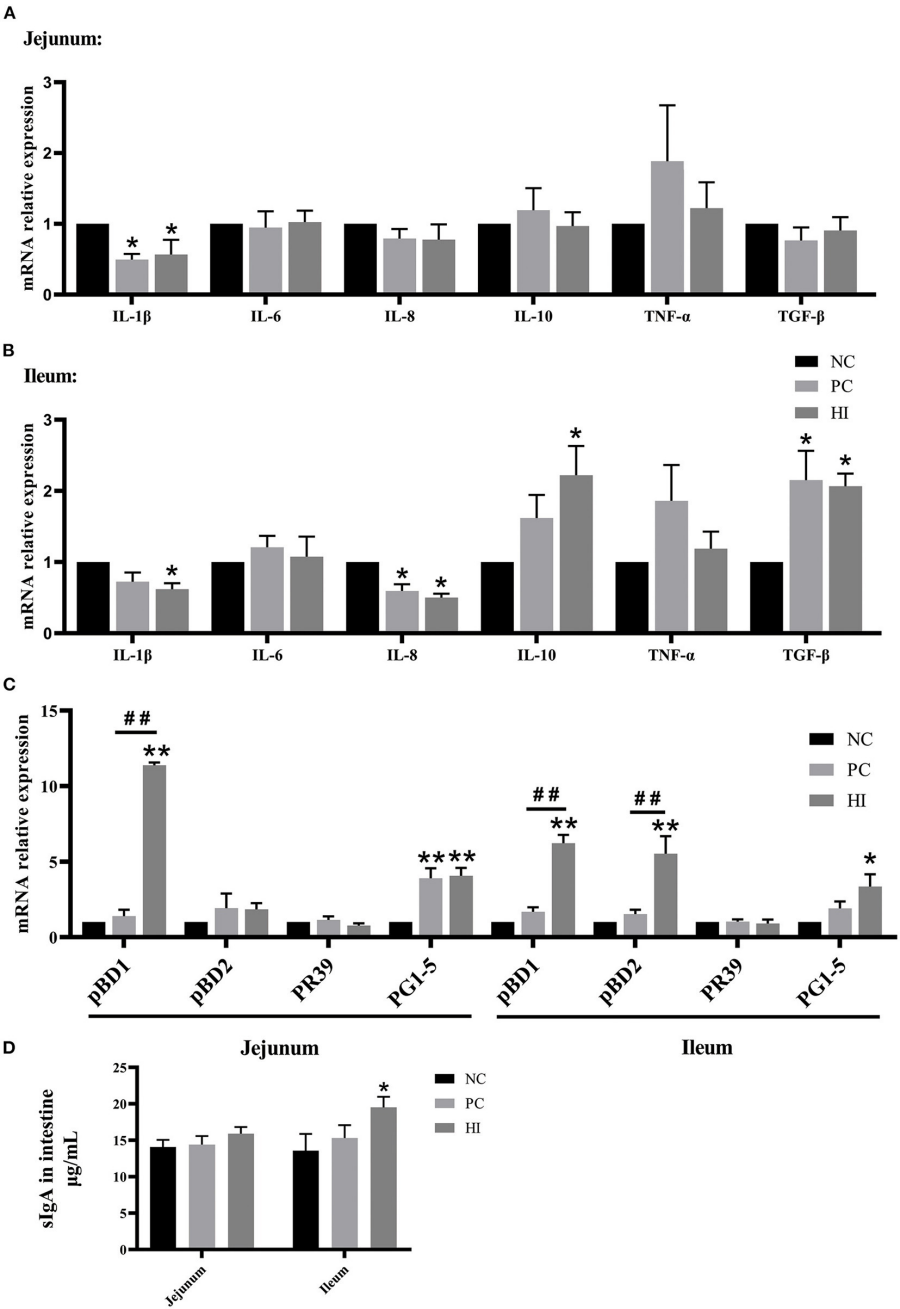
Effect of dietary *Hermetia illucens* larvae on intestinal immunity of weaned pigs. Real-time quantitative PCR was performed to determine the relative mRNA expression levels of *IL-1*β, *IL-6, IL-8, IL-10, TNF-*α, and *TGF-*β in the jejunum **(A)** and ileum **(B)**. **(C)** Real-time quantitative PCR was performed to determine the relative mRNA expression levels of *pBD1, pBD2*, proline-arginine rich 39-amino acid peptide (*PR39*), and *PG1-5* in the jejunum and ileum. **(D)** Concentration of sIgA in the jejunum and ileum determined using ELISA. All data are expressed as the mean ± SEM (*n* = 6). *p < 0.05, **p < 0.01 compared with NC, ^##^*p* < 0.01 HI compared with PC.

Regarding intestinal immunity, there was a decrease (*p* < 0.05) in interleukin-1β (*IL-1*β) expression in the jejunum and *IL-8* expression in the ileum, and an increase (*p* < 0.05) in transforming growth factor-β (*TGF-*β) expression in the ileum of pigs in the PC and HI groups compared with those of pigs in the NC group (Figures 3A,B). Additionally, there was a decrease (*p* < 0.05) in *IL-1*β expression and an increase (*p* < 0.05) in *IL-10* expression in the ileum of pigs in the HI group compared with those of pigs in the NC group. Furthermore, there was an increase (*p* < 0.01) in porcine β-defensin 1 (*pBD1*) expression in the jejunum and ileum, and an increase (*p* < 0.01) in *pBD2* expression in the ileum of pigs in the HI group compared with those of pigs in the NC and PC groups (Figure 3C). Compared with the NC group, there was an increase (*p* < 0.01) in protegrin 1-5 (*PG1-5*) expression in the jejunum of pigs in the PC and HI groups. Moreover, there was an increase (*p* < 0.05) in *PG1-5* expression and sIgA concentration in the ileum of pigs in the HI compared with those of pigs in the NC group (Figure 3D).

### Effect of *H. illucens* on the TLR2-NF-κB/MAPK Signaling Pathway and Histone Acetylation Modification in the Intestine of Weaned Pigs

**Figure 4:**
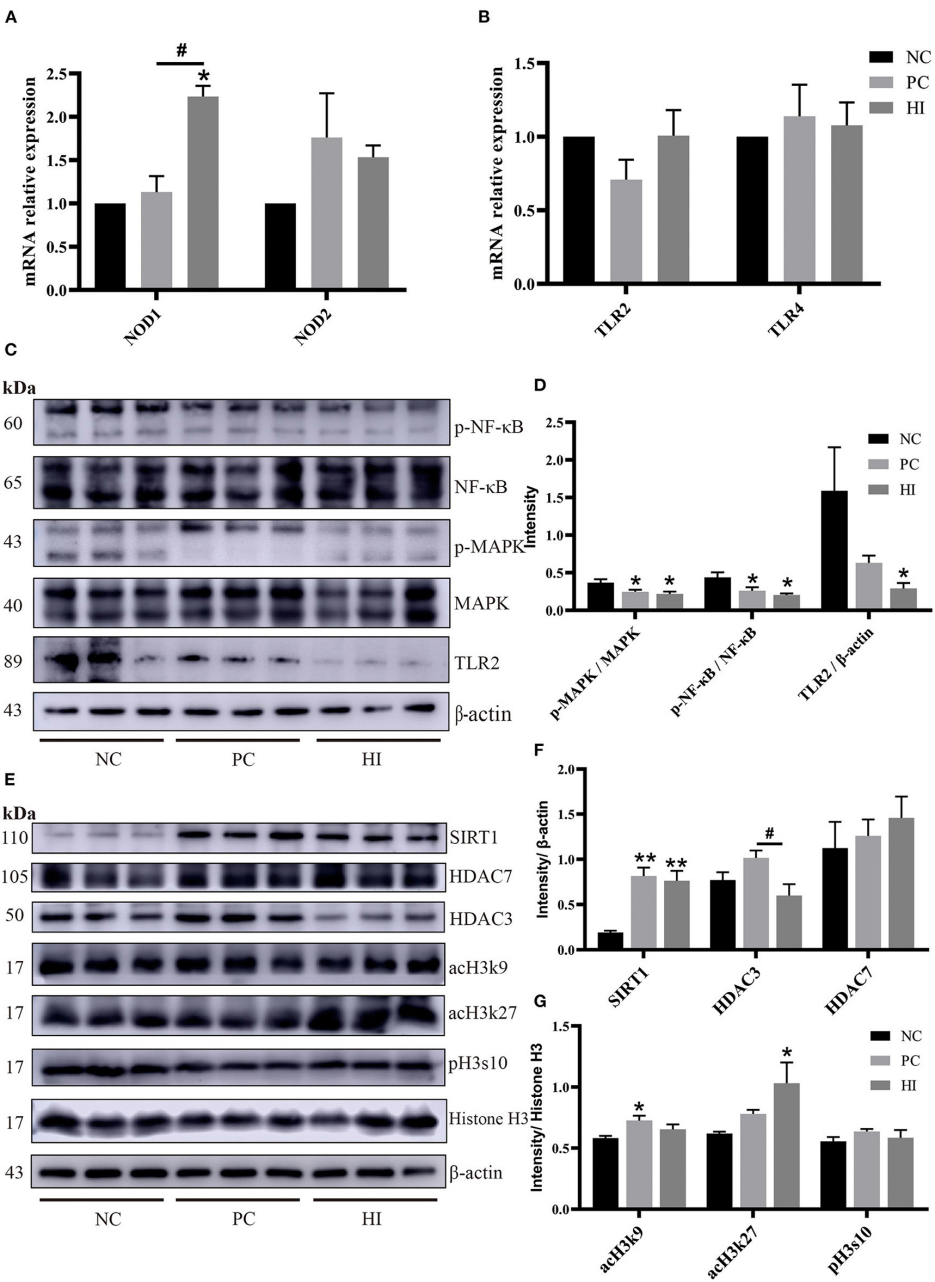
Effect of dietary *Hermetia illucens* larvae on TLR2-NF-κB/MAPK signaling pathway and histone acetylation modification in weaned pigs. Real-time quantitative PCR was performed to determine the relative mRNA expression levels of *NOD1, NOD2* **(A)**, *TLR2*, and *TLR4* **(B)** in the jejunum and ileum. **(C)** Relative protein expression levels of p-NFκB, NFκB, p-MAPK, MAPK, TLR2, and β-actin in the ileum were determined using western blot. **(D)** Statistical analysis of the data in **(C)**. **(E)** Western blotting was performed to determine the relative protein expression levels of SIRT1, HDAC7, HDAC3, acH3k9, acH3k27, histone H3, β-actin, and phosphorylation of histone H3 at serine S10 (pH3s10) in the ileum. **(F,G)** Statistical analysis of the data in (E). All data are expressed as the mean ± SEM (protein expression data *n* = 3, other data *n* = 6). *p < 0.05, **p < 0.01 compared with NC, ^#^*p* < 0.05 HI compared with PC.

To explore the possible mechanisms of HI in immune responses, changes in histone acetylation modification, and the expression of nucleotide-binding oligomerization domains (NODs), toll-like receptor (TLR), NF-κB, and MAPK in the ileum, were examined. Compared with the NC and PC groups, there was an increase (*p* < 0.05) in *NOD1* expression and a decrease (*p* < 0.05) in TLR2 protein expression in the ileum of pigs in the HI group (Figures 4A–D). Additionally, there was a decrease (*p* < 0.05) in p-NF-κB/NF-κB and p-MAPK/MAPK ratio and an increase (*p* < 0.05) SIRT1 protein expression in the ileum of pigs in the HI and PC groups compared with those of pigs in the NC group. Moreover, there was an increase (*p* < 0.05) in HDAC3 protein expression in the ileum of pigs in the PC group compared with those of pigs in the HI and NC groups (Figures 4E,F). Compared with the HI and NC groups, there was an increase (*p* < 0.05) in acH3k9 protein expression in the ileum of pigs in the PC group. Additionally, there was an increase (*p* < 0.05) in acH3k27 protein expression in the ileum of pigs in the HI group compared with those of pigs in the PC and NC groups (Figures 4E,G).

### Effect of *H. illucens* on the Colon Microbiota and SCFAs of Weaned Pigs

**Figure 5:**
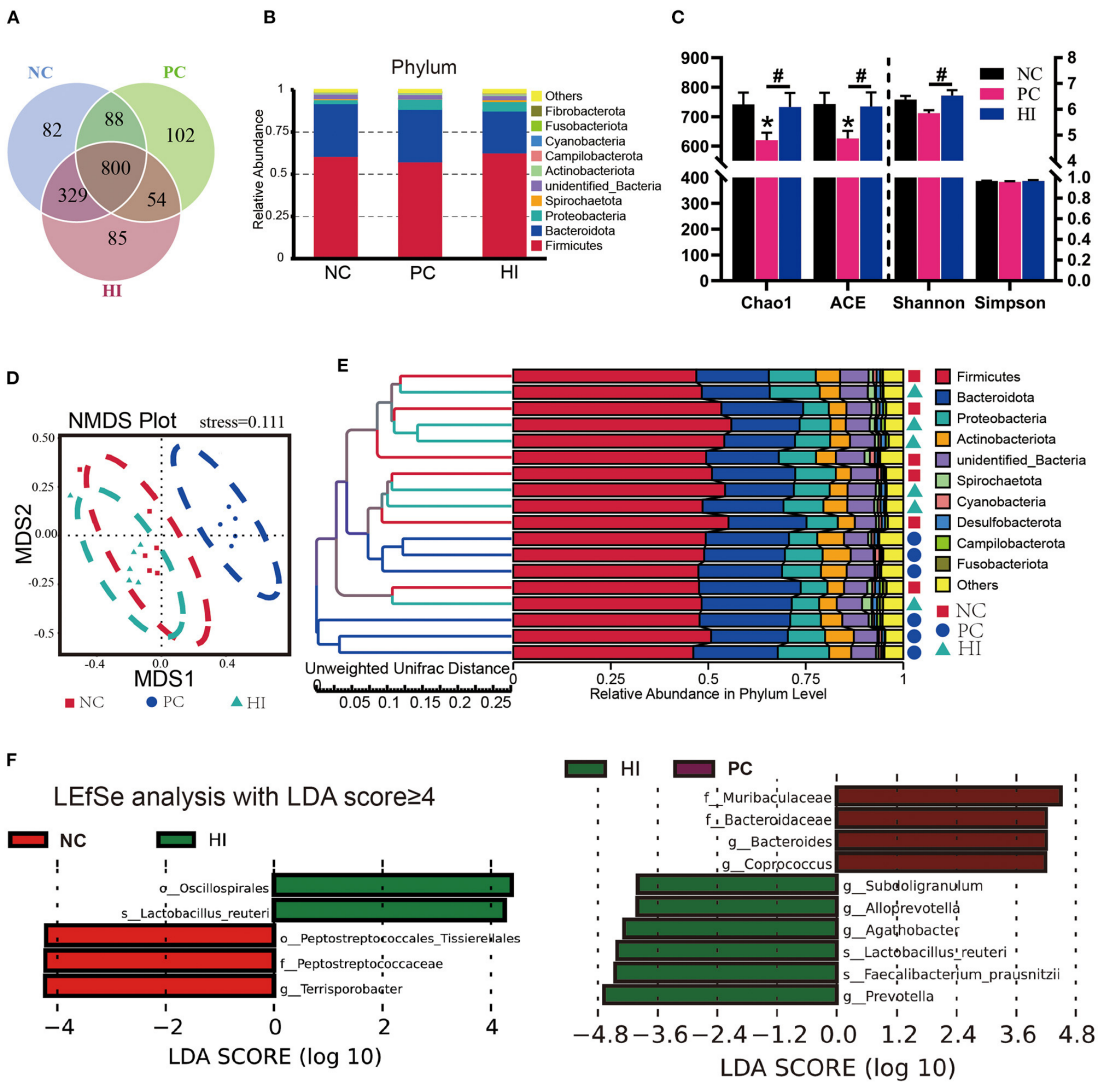
Effect of dietary *Hermetia illucens* larvae on the microbial communities in the colonic contents of weaned pigs. **(A)** Venn diagram illustrating common and special OTUs distributed among the three groups. **(B)** The phylum-level species histogram shows the top 10 phylum-level species. **(C)** Species diversity and homogeneity were evaluated using Chao1, ACE, Shannon's, and Simpson's indices. **(D)** OUTs-based NMDS plot. **(E)** UPGMA clustering was conducted based on unweighted unifrac distance. **(F)** Significantly different biomarkers in the three groups. *n* = 6. *p < 0.05 compared with NC, ^#^*p* < 0.05 HI compared with PC.

**Figure 6:**
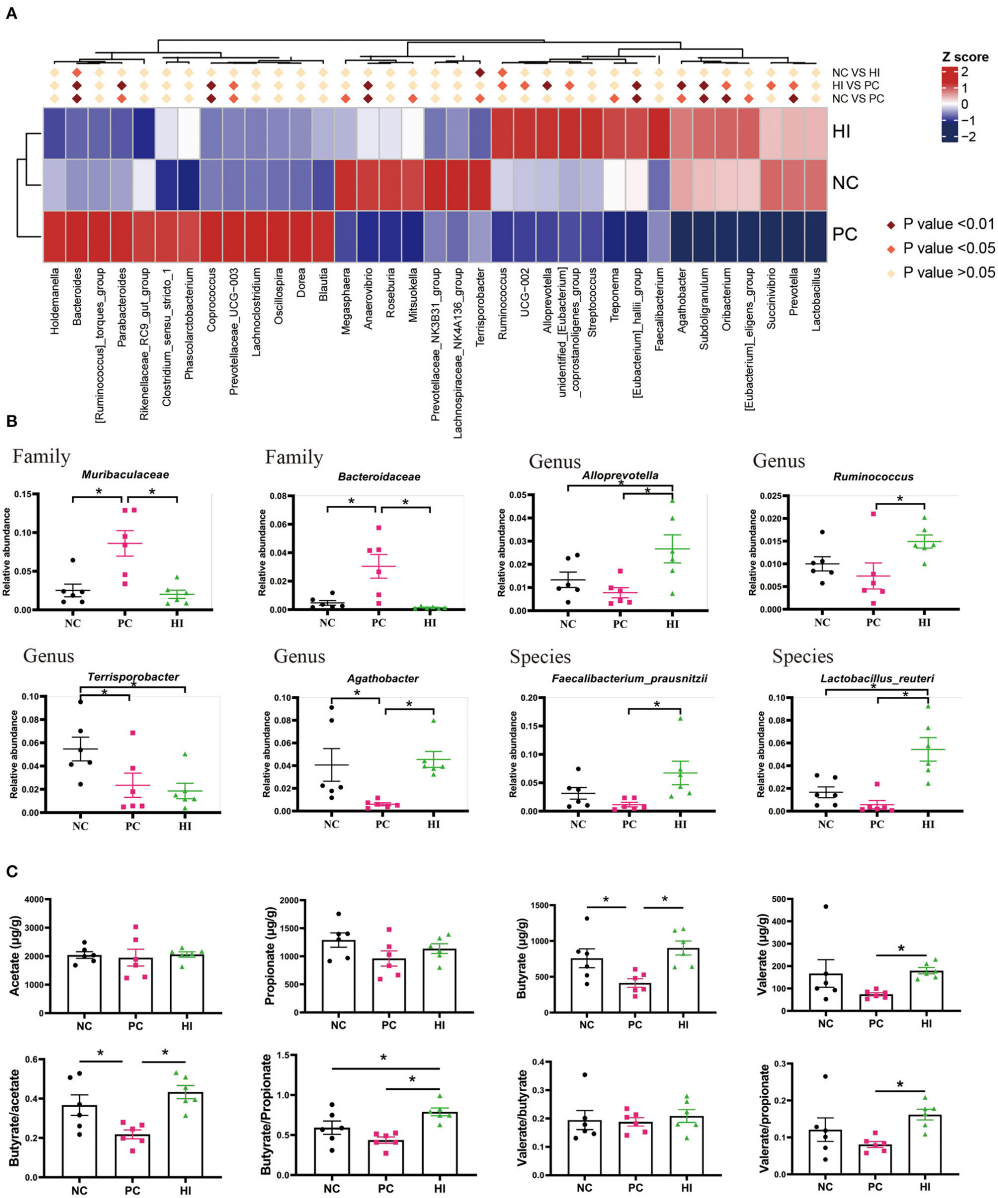
Effects of dietary *Hermetia illucens* larvae on the microbial composition at different levels and the concentration of SCFAs in the colonic content of weaned pigs. **(A)** Heat maps of different species at the genus level. **(B)** Relative abundance of intestinal microbiota at the family, genus, and species level. **(C)** Acetate, propionate, butyrate, and valerate concentration of the colonic contents were determined using GC-MS, while butyrate/acetate, butyrate/propionate, valerate/butyrate, and valerate/propionate ratios were calculated. *n* = 6. *p < 0.05.

The microbial composition of the colonic digesta in response to HI treatment was determined using 16S rRNA Illumina MiSeq sequencing. Figure 5A shows the distributions of common and specific OTUs among the three groups. At the phylum level, Firmicutes, Bacteroidetes, and Proteobacteria were the three most abundant bacteria in all groups (Figure 5B). Compared with the NC and HI group, there was a decrease (*p* < 0.05) in Chao1 and ACE indices of the colonic content of pigs in the PC group. Additionally, there was a decrease (*p* < 0.05) in the Shannon's index of the colonic content of pigs in the PC group compared with those of pigs in the HI group (Figure 5C). As shown in the NMDS plot (Figure 5D), samples from the PC group formed a distinct cluster from those of the other groups. A phenetic tree of the three groups was constructed based on unweighted unifrac distance using UPGMA clustering method (Figure 5E). Furthermore, LEFSe analysis identified statistically different bacteria between the three groups (Figure 5F). Petostreptococcales Tissierellales, *Peptostreptococcaceae*, and *Terrisporobacter* were more abundant in the NC group, whereas *Muribaculaceae, Bacteroidaceae*, Bacteroides, and *Coprococcus* were more abundant in the PC group. Oscillospirales, *Subooligranulum, Alloprevotella, Agathobacter, Lactobacillus reuteri, Faecalibacterium prausnitzii*, and *Prevotella* were more abundant in the HI group. The relative abundance of different genera at the genus level is shown in Figure 6A. Furthermore, univariate statistical analysis indicated an increase (*p* < 0.05) in the abundance of *Muribaculaceae* and *Bacteroidaceae* and a decrease (*p* < 0.05) in the abundance of *Agathobacter* in the colonic content of pigs in the PC group compared with those of pigs in the NC and HI group (Figure 6B). Compared to the NC group, there was a decrease (*p* < 0.05) in the abundance of *Terrisporobacter* in the colonic content of pigs in the HI and PC groups. In addition, there was an increase (*p* < 0.05) in the abundance of *Ruminococcus* and *Faecalibacterium prausnitzii*, and *Alloprevotella* and *Lactobacillus reuteri* in the colonic content of pigs in the HI group compared with those of pigs in the PC group, and NC and PC groups, respectively.

Regarding SCFAs, there was a decrease (*p* < 0.05) in the concentration of butyrate and butyrate/acetate ratio in pigs in the PC group compared with those of pigs in the NC and HI groups (Figure 6C). There was also an increase in the valerate concentration and valerate/propionate ratio in pigs in the HI group compared with those of pigs in the PC group, and NC and PC groups, respectively.

### Correlation Analysis Between Dominant Bacteria, SCFAs, Barrier Function Factor, and Immune Factor

**Figure 7:**
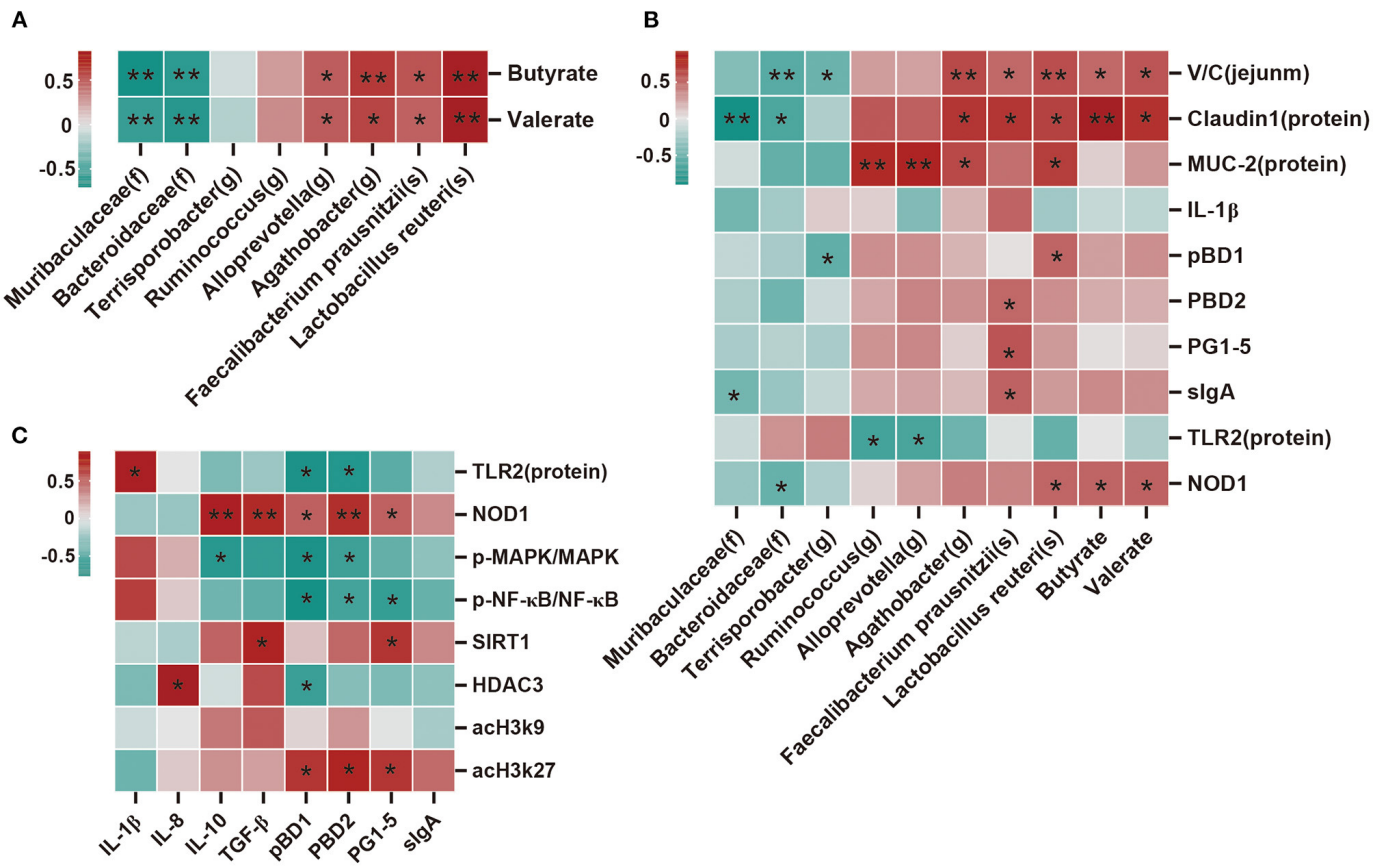
Correlation analysis of the dominant bacteria, SCFAs, barrier function factor, and immune factor. **(A)** Correlation analysis between microbial species and SCFAs. **(B)** Correlation analysis between microbial species, SCFAs, and measured parameters. **(C)** Correlation analysis between the immune index and immunomodulatory factor. Except for microbial species, SCFAs, and V/C (ratio of villi height to crypt depth), all other indicators belong to the ileum. There are indicators for mRNA expression and protein expression data, and protein expression data was selected for analysis. Protein expression data *n* = 3, other data *n* = 6. Red represents positive correlation and green represents negative correlation (*p < 0.05, **p < 0.01).

Spearman's correlation analysis was performed to determine whether there was any relationship between microbial species, SCFAs, and expression levels of barrier function factor and immunomodulatory factors. As shown in Figure 7A, butyrate and valerate were positively correlated (*p* < 0.05) with *Alloprevotella, Agathobacter, Faecalibacterium prausnitzii*, and *Lactobacillus reuteri*, but negatively correlated (*p* < 0.05) with *Muribaculaceae* and *Bacteroidaceae*. Moreover, *Muribaculaceae* was negatively correlated (*p* < 0.05) with claudin1 and sIgA; *Bacteroidaceae* was negatively correlated (*p* < 0.05) with V/C, claudin1, and NOD1; and *Terrisporobacter* was negatively correlated (*P* < 0.05) with V/C and *pBD1*. Conversely, *Agathobacter* was positively (*p* < 0.05) correlated with V/C, claudin1, MUC-2; *Faecalibacterium prausnitzii* was positively (*p* < 0.05) correlated with V/C, claudin1, *IL-1*β, *pBD2, PG1-5*, and sIgA; and *Lactobacillus reuteri* was positively (*p* < 0.05) correlated with V/C, claudin1, MUC-2, *pBD1*, and NOD1. Additionally, *Ruminococcus* and *Alloprevotella* were positively (*P* < 0.05) correlated with MUC-2, but negatively correlated (*P* < 0.05) with TLR2 (Figure 7B). NOD1 was positively correlated (*p* < 0.05) with *IL-10, TGF-*β, *pBD1, pBD2*, and *PG1-5*; SIRT1 was positively correlated (*p* < 0.05) with *TGF-*β and *PG1-5*; and acH3k27 was positively correlated (*p* < 0.05) with *pBD1, pBD2*, and *PG1-5*. In contrast, p-MAPK/MAPK was negatively correlated (*p* < 0.05) with *IL-10, pBD1, pBD2* and p-NF-κB/NF-κB was negatively correlated (*p* < 0.05) with *pBD1, pBD2*, and *PG1-5*. HDAC3 was positively correlated (*p* < 0.05) with IL-8, but negatively correlated (*p* < 0.05) with *pBD1* (Figure 7C).

## Discussion

**Figure 8:**
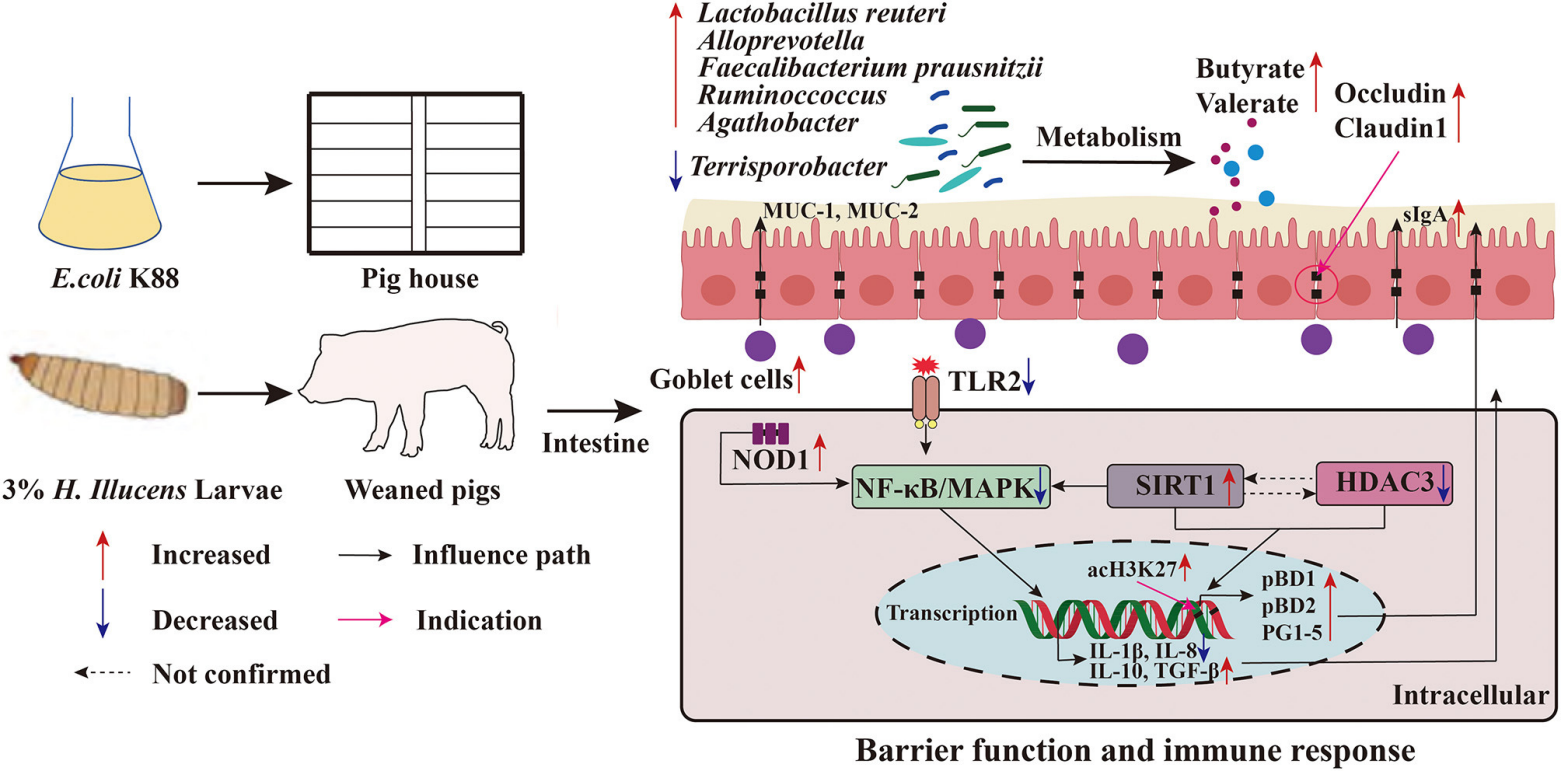
Function model of the proposed *Hermetia illucens* larvae meal. The red arrow indicates an increase in the value of the detected parameter compared to NC or PC, and blue indicates a decrease.

Early weaning (3–4 weeks) can cause nutritional and environmental stress in piglets, which can lead to impaired gut function, reduced immunity, inefficient feed utilization, and retarded growth (24). Optimal intestinal barrier function is essential for nutrient absorption and delivery. This study showed that 100% replacement of FM with HI significantly improved the growth performance and reduced the diarrhea incidence of weaned pigs. Moreover, HI addition improved the intestinal barrier function by regulating goblet cell population, mucin production, expression of TJ proteins and AMPs, cytokine levels, and intestinal microbiota (Figure 8). Furthermore, the findings of this study showed that HI regulated the expression of intestinal cytokines through the TLR2-NF-κB/MAKP signaling pathway and promoted histone acetylation modification by increasing SIRT1 expression and decreasing HDAC3 expression, regulating the expression of AMPs. These positive results suggest that HI could be used as a protein source in pig nutrition.

Over the last few decades, studies have shown that diets high in zinc oxide (1,000–3,000 mg zinc/kg) can reduce the incidence of diarrhea and improve the growth performance of weaned pigs (25, 26). Similarly, this study showed that dietary supplementation with 1,445 mg zinc/kg zinc oxide increased the ADFI and ADG of weaned pigs during day 1–14 of the study. However, zinc oxide use is gradually decreasing because of its negative effects on animals and the environment. Previous studies have shown that the addition of HI in diets at appropriate levels can improve the growth performance of Chinese soft-shelled turtles, chickens, and fattening pigs (6–8). A recent study showed that feeding small amounts of live *H. illucens* larvae had no adverse effect on the growth performance of weaned pigs (27). Surprisingly, the pigs that were fed HI-supplemented diet showed increased ADG and ADFI from day 1 to 14, similar to those that were fed zinc oxide-supplemented diets. In addition, feeding HI-supplemented diet reduced diarrhea incidence from 28.17–32.76% in the control group to 15.48–20.11%; however, pigs fed a zinc oxide-supplemented diet had the lowest diarrhea incidence rate (1.98–2.30%). These findings indicated that HI improved the growth performance of weaned pigs in the first 2 weeks post-weaning and reduced the diarrhea incidence in weaned pigs during the experimental period.

Furthermore, the effect of HI on parameters and genes involved in intestinal health, such as goblet cells, MUC-1, MUC-2, and several intestinal immunity-related genes, was examined in this study. The primary function of intestinal goblet cells is to form and secrete mucus, which plays an important role in protecting the intestinal epithelial barrier from pathogen invasion (28). Among other physiological effects, ETEC K88 can cause damage to the mucus layer of the intestinal mucosa. However, this study showed that feeding HI-supplemented diet protected the pigs against ETEC K88 strain by increasing the mucus layer thickness and goblet cells. Moreover, there was an increase in the expression of MUC-1 and MUC-2 in the intestine of pigs fed HI-supplemented diet, thus validating the increase in mucus layer thickness of these pigs, and this improvement is better than zinc oxide-supplemented diets. MUC-2 plays an essential role in the tissue mucus layer and in mucosal repair (29). Previous studies have shown that 4% addition of HI increased *MUC-1* expression in the intestines of fattening pigs and weaned pigs (11, 14). These data suggest that HI improved intestinal barrier function through regulating mucin protein production in the intestine.

TJ proteins are involved in the integrity of tight junction structures by binding to the cellular skeleton and are thought to be essential regulators of paracellular permeability (30). Previous studies have shown that ETEC K88 can reduce the expression of TJ proteins and cause intestinal barrier dysfunction (31). In this study, feeding HI-supplemented diet improved the intestinal morphology of weaned pigs at 28 days after weaning. Moreover, addition of HI increased claudin-1 expression in the jejunum and occludin expression in the ileum, which was higher than the expression levels in pigs fed zinc oxide-supplemented diets. A previous study reported that 4% addition of HI enhanced *ZO-1, occludin*, and *claudin-2* expression in the ileum of weaned pigs (14). However, the expression of *ZO-1* contradicted the results of the present study, which could be attributed to the differences in study environment, such as the presence of ETEC K88. Overall, feeding HI-supplemented diets improved jejunum morphology and the expression of TJ protein, which improved intestinal epithelial barrier function in weaned pigs.

Cytokines play essential roles in the integrity of the intestinal barrier. Pro-inflammatory cytokines often disrupt TJs, whereas anti-inflammatory cytokines protect the epithelial barrier function (32). Improved growth performance and reduced diarrhea incidence have been linked to improved immune function in weaned pigs. This study showed HI addition in the diets of piglets caused a decrease in the expression of *IL-1*β and *IL-8*, and an increase in *IL-10* and *TGF-*β expression in the intestine, similar to those fed zinc oxide-supplemented diets. Recent studies have shown that feeding diets containing 2 or 4% HI can decrease *TNF-*α and *IFN-*γ expression and increase *IL-10* expression in the intestinal mucosa of weaned pigs or fattening pigs (11, 14). These findings suggest that HI improved growth performance and reduced diarrhea incidence by improving intestinal immunity and barrier function.

As an important component of specific immune response, antibacterial proteins are the first line of defense against intestinal toxins and pathogenic microorganisms (33). This study showed there was an increase in ileal sIgA secretion but not jejunal secretion in pigs fed diet containing HI. We speculate this may be because a certain component of HI accumulates in the terminal ileum and exerts a strong stimulating effect. Studies have established that insects are one of the major sources of AMPs (34). Previous studies have reported that AMPs can alleviate epithelial barrier damage in the intestinal epithelial cells of diarrheic weaned pigs and infected pigs (22, 35). However, whether feeding HI-supplemented diet can promote the secretion of AMPs in the intestinal mucosa of weaned pigs has not been examined. This study showed HI-supplemented diets increased the expression of *pBD1, pBD2*, and *PG1-5* in the intestine, and zinc oxide-supplemented diets increased the expression of *PG1-5* in the intestine. Overall, this study showed that HI improved the intestinal immune barrier function in weaned pigs by regulating sIgA secretion, and the expression of cytokines and AMPs, and the improvement is better than that seen in zinc oxide-supplemented diets.

The regulatory mechanism of intestinal immunity is complex and involves multiple signaling pathways. TLRs are innate immune receptors that recognize conserved microbial structures and host alarm proteins and are involved in regulating inflammatory responses (36, 37). NOD1 and NOD2 rely on well-orchestrated downstream signaling networks that involve the MAPK and NF-κB pathways for regulating host defense and tissue homeostasis. The NF-κB and MAPK signaling pathways are two classical pathways associated with immune regulation and play important roles in processes such as inflammation and cell survival (38, 39). Notably, the present study showed a HI-supplemented diet inhibited the TLR2-NF-κB/MAPK signaling pathway and increased *NOD1* expression. A zinc oxide-supplemented diet also inhibited NF-κB/MAPK activation. Next, we investigated the mechanisms by which HI induced AMPs expression. Histone acetylation opens the chromatin structure and facilitates the binding of transcription factors to transcription sites to promote gene transcriptional expression. The elevated levels of acetylated histone H3 lysine 27 (acH3k27), acetylated histone H3 lysine 56 (acH3k56), and pH3s10 in the promoter region are typical markers of transcriptional activation of AMPs. Inhibition of HDAC regulates the modification mode of histone H3 (acH3k9, acH3k27, pH3s10, etc.) in the promoter region of AMPs, which specifically increases the expression of intestinal AMPs and enhances their ability to combat bacterial infections (15–17, 40). Sirtuins are members of HDAC (40). Recent studies have shown that SIRT1 plays an important role in maintaining intestinal mucosal barrier integrity (41, 42). However, SIRT1 and HDAC interact to regulate the expression of intestinal AMPs (43, 44). In addition, SIRT1 inhibits the activation of the NF-κB/MAPK signaling pathway and participates in immune regulation (40, 45). This study showed that HI-supplemented diets increased the level of acH3k27 in the promoter region, and zinc oxide-supplemented diets increased the level of acH3k9 in the promoter region. These data suggest that the increase in intestinal AMP expression in pigs fed HI-supplemented diet was associated with increased levels of SIRT1 and acH3k27, but zinc oxide-supplemented diets were associated with increased levels of SIRT1 and acH3k9. Overall, feeding HI-supplemented diet inhibited the activation of the TLR2-NF-κB/MAPK pathway and promoted histone acetylation modification, enhancing the intestinal immune regulation in weaned pigs.

Epithelial barrier impairment and immune disturbances are usually associated with disruption of the host gut microbial composition during pathogenic infection (22). This study showed that feeding HI-supplemented diet did not significantly influence intestinal microbiota diversity. However, there was an increase in the relative abundance of some species, such as *Lactobacillus reuteri* in the intestines of pigs fed HI-supplemented diet. Our previous studies found that *Lactobacillus reuteri* promoted the metabolism of intestinal amino acids and improved the intestinal barrier function in weaned pigs (46, 47). Additionally, there was an increase in the relative abundance of *Alloprevotella, Ruminococcus, Agathobacter*, and *Faecalibacterium prausnitzii* in the colon of pigs fed HI-supplemented diets. Notably, *Ruminococcus* and *Faecalibacterium prausnitzii* are important butyrate-producing bacteria (48–50). In the present study, there was a decrease in the abundance of *Terrisporobacter*, which is an emerging anaerobic pathogen (51), in the colon of pigs fed HI-supplemented diets. Regulating the intestinal microbiota by HI may be related to its high AMP and fatty acid content. Insect AMPs have broad-spectrum antimicrobial properties, and lauric acid (C12:0) is particularly effective for the inhibition of gram-positive bacteria (5).

SCFAs have several beneficial effects on the dynamic balance of the colon by promoting energy metabolism of epithelial cells and stimulating immune development (49, 52). SCFAs provide energy and can lower intestinal pH and inhibit the proliferation of pathogenic bacteria (53). Previous studies have shown that butyrate is a major source of energy for colonic epithelial cells and has anti-inflammatory effects (54). In this study, there was an increase in butyrate to propionate ratio in the colon of pigs fed HI-supplemented diets. These results indicate that HI-supplemented diets had no negative effects on intestinal microbiota and their metabolic activities, which was contrary to observations in pigs in the PC group. Moreover, correlation analysis revealed that *Lactobacillus reuteri*, which was high in pigs fed HI-supplemented diets, showed a strong positive correlation with butyrate and valerate, but strong negative correlation with *Muribaculaceae* and *Bacteroidaceae*. These results suggest that *Lactobacillus reuteri, Muribaculaceae*, and *Bacteroidaceae* may have a regulatory effect on SCFA production and intestinal barrier function.

In this study, *Lactobacillus reuteri* was positively correlated with SCFAs (butyrate and valerate), and intestinal barrier function factors, including *NOD1*, but negatively correlated with TLR2. However, *Muribaculaceae* and *Bacteroidaceae* had the opposite relationship with SCFAs, intestinal barrier function factors, and TLR2. We speculated that *Lactobacillus reuteri*, and SCFAs may improve the intestinal barrier function of weaned pigs by mediating TLR2 and NOD1, whereas *Muribaculaceae* and *Bacteroidaceae* may inhibit this pathway, but the potential mechanism needs to be further studied. Additionally, TLR2, p-MAPK/MAPK, and P-NF-KB/NF-κB were negatively correlated with the expression of AMPs, *IL-10*, and *TGF-*β; whereas NOD1, SIRT1, and acH3k27 were positively correlated with *IL-10, TGF-*β, and AMPs. This suggests there may be an interaction between cytokines regulated by the TLR2-NF-κB/MAPK pathway and the expression of AMPs regulated by SIRT1 and HDAC3, but further studies are needed to clarify the potential mechanism.

## Conclusion

A 100% replacement of FM with HI improved the growth performance of weaned pigs in the first 2 weeks after weaning and reduced the incidence of diarrhea after weaning in the environment of ETEC K88. Moreover, dietary HI improved intestinal barrier function and microflora. Additionally, HI-supplemented diet improved intestinal immune homeostasis by promoting histone acetylation and inhibiting the activation of the TLR2-NF-κB/MAPK signaling pathway in weaned pigs.

## Data Availability Statement

The datasets presented in this study can be found in online repositories. The names of the repository/repositories and accession number(s) can be found below: https://www.ncbi.nlm.nih.gov/, PRJNA782794.

## Ethics Statement

The experiments were conducted in accordance with the Chinese guidelines for animal welfare and experimental protocols, and all animal experimental procedures were approved by the Animal Care and Use Committee of Guangdong Academy of Agricultural Sciences (Guangzhou, China). Permit Number: GAASIAS-2020-039.

## Author Contributions

HY and LW designed the experiments. QT and EX drafted the manuscript and sample analysis. MX, ZW, SC, and SH conducted the experiments and data curation. QW, YX, FW, and GY verified the validity of experiment and checked the results. ZJ supervised the entire study. All authors have read and agreed to the published version of the manuscript.

## Funding

This study was funded by the China Agriculture Research System of MOF and MARA, the Outstanding Talents Training Program of Guangdong Academy of Agricultural Sciences (R2018PY-JC001), a special fund for scientific innovation strategy construction of the high-level Academy of Agriculture Science (R2016YJ-YB2003, R2019PY-QF005, R2018QD-068), the project of swine innovation team in Guangdong Modern Agricultural Research System (2021KJ126), and Independent Research and Development Projects of Maoming Laboratory (2021ZZ003).

## Conflict of Interest

FW and GY were employed by the company Guangzhou AnRuiJie Environmental Protection Technology Co., Ltd. The remaining authors declare that the research was conducted in the absence of any commercial or financial relationships that could be construed as a potential conflict of interest.

## Publisher's Note

All claims expressed in this article are solely those of the authors and do not necessarily represent those of their affiliated organizations, or those of the publisher, the editors and the reviewers. Any product that may be evaluated in this article, or claim that may be made by its manufacturer, is not guaranteed or endorsed by the publisher.
