# Supplementary material for: Dietary Hermetia illucens Larvae Meal Improves Growth Performance and Intestinal Barrier Function of Weaned Pigs Under the Environment of Enterotoxigenic Escherichia coli K88
Source: Front Nutr. 2022 Jan 18;8:812011. doi: 10.3389/fnut.2021.812011 (PMC8805673; doi:10.3389/fnut.2021.812011)
Supplement: Supplementary file 1 [file Data_Sheet_1.docx]

Supplementary Material

# Supplementary Data

The original data can be downloaded via this link: <https://www.jianguoyun.com/p/Dfb3818Qs5iFChjqv6AE>
